# Targeting fatty acid oxidation via Acyl-CoA binding protein hinders glioblastoma invasion

**DOI:** 10.1038/s41419-023-05813-0

**Published:** 2023-04-29

**Authors:** Ceren Duman, Barbara Di Marco, Ekaterina Nevedomskaya, Berk Ulug, Ralf Lesche, Sven Christian, Julieta Alfonso

**Affiliations:** 1 Department of Clinical Neurobiology, University Hospital Heidelberg and German Cancer Research Center (DKFZ), Heidelberg, Germany; 2 Bayer Research & Innovation Center, Cambridge, MA USA; 3 Present Address: NUVISAN ICB GmbH, Berlin, Germany

**Keywords:** Cancer metabolism, Cell invasion

## Abstract

The diffuse nature of Glioblastoma (GBM) tumors poses a challenge to current therapeutic options. We have previously shown that Acyl-CoA Binding Protein (ACBP, also known as DBI) regulates lipid metabolism in GBM cells, favoring fatty acid oxidation (FAO). Here we show that ACBP downregulation results in wide transcriptional changes affecting invasion-related genes. In vivo experiments using patient-derived xenografts combined with in vitro models demonstrated that ACBP sustains GBM invasion via binding to fatty acyl-CoAs. Blocking FAO mimics ACBP^KD^-induced immobility, a cellular phenotype that can be rescued by increasing FAO rates. Further investigation into ACBP-downstream pathways served to identify Integrin beta-1, a gene downregulated upon inhibition of either ACBP expression or FAO rates, as a mediator for ACBP’s role in GBM invasion. Altogether, our findings highlight a role for FAO in GBM invasion and reveal ACBP as a therapeutic vulnerability to stall FAO and subsequent cell invasion in GBM tumors.

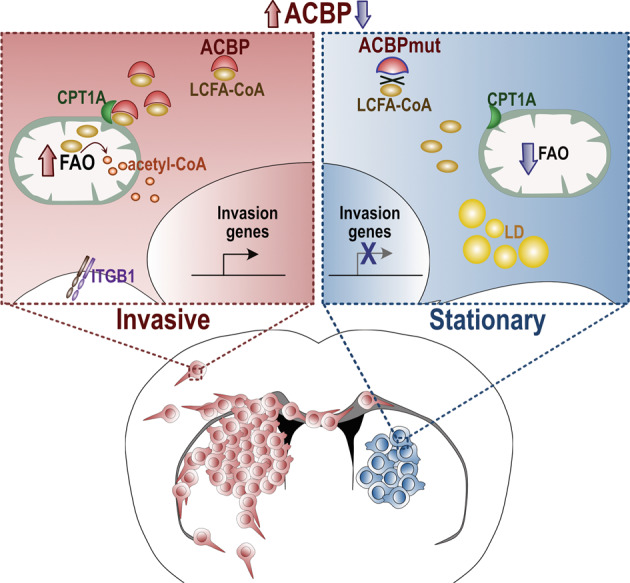

## Introduction

Glioblastoma multiforme (GBM) is the most aggressive and common type of glioma in adults [1]. Despite receiving the standard of care, GBM patients have a poor prognosis with a median overall survival of only 12–15 months mainly due to the inevitable recurrence of these tumors [2]. Indeed, the diffuse nature of GBM tumors poses a significant threat of relapse after surgical removal of the tumor mass [3]. Current therapies consist largely of surgical resection followed by antiproliferative treatments directed at fast-dividing cells; however, relatively quiescent cell populations such as cancer stem-like and infiltrating cells [4, 5] might not be targeted by irradiation and/or chemotherapy. These chemo-radioresistant populations that escape surgical resection can eventually act as tumor-initiating cells and regrow the tumors [6, 7]. Hence, a better understanding of molecular pathways involved in GBM invasion could help discover novel vulnerabilities.

Like other cancer types, one key hallmark of brain tumors is altered cellular metabolism. Metabolic adaptations such as increased aerobic and anaerobic glycolysis and enhanced synthesis and utilization of lipids, amino acids and nucleotides are common in glioma cells [8]. One obvious outcome of these metabolic rearrangements is increased energy and biomass generation to support tumor growth and invasion [9]. We and others have previously shown that fatty acid oxidation (FAO) is a major energetic pathway in GBM cells, and that active FAO induces fast tumor growth via affecting cell proliferation rates [10–13]. Currently, little is known about lipid metabolic alterations involved in glioma invasion and only a few studies in other cancer types have suggested a role for FAO in tumor cell invasion [14, 15].

In order to be oxidized, free fatty acids need first to be converted to fatty acyl-CoAs and then shuttled inside the mitochondrial matrix [16]. Acyl-CoA Binding Protein (ACBP, previously named DBI) is a small cytosolic protein that binds with high affinity and specificity to medium and long-chain fatty acyl-CoAs (M-LCACoA) [17] and transports them across the cytoplasm towards mitochondria, thereby incrementing FAO [18–20]. We have previously shown that ACBP is highly expressed in GBM cells and targeting this protein in preclinical GBM models extends the survival of the experimental animals [11]. In this study, we used an unbiased screen to investigate further the cellular and molecular pathways involved in ACBP-mediated tumor aggressivity. Our results from in vitro and in vivo models showed a dual function of ACBP in GBM cell proliferation and invasion, through its ability to mediate fatty acid metabolism. Considering the culpability of aggressive tumor cell invasion in the low success rate of current GBM therapies, targeting ACBP could potentially damage both proliferative and migratory capacity of these devastating tumors.

## Results

### Dysregulated transcriptional pathways in ACBP^KD^ cancer cells

**Fig. 1:**
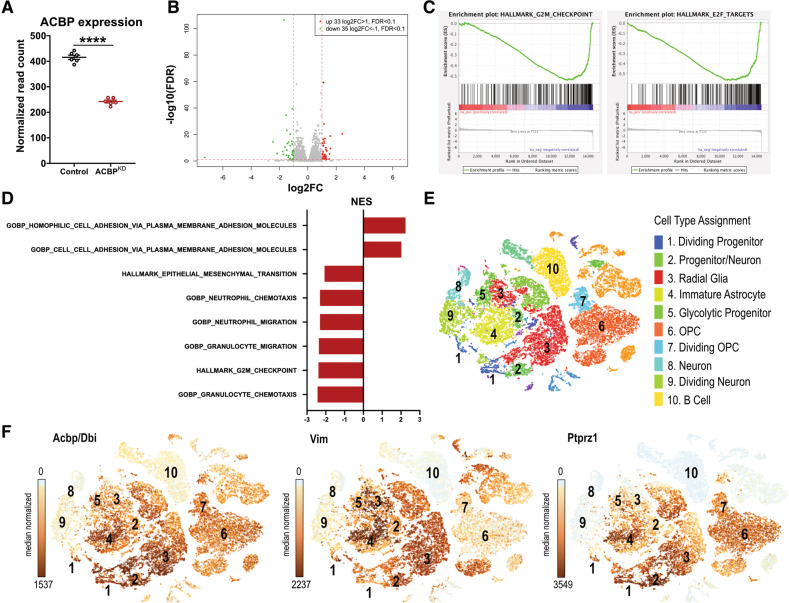
ACBP downregulation affects cell migration and invasion transcriptional pathways in GBM cells. **A** LN229 GBM cells were transduced with lentiviruses expressing either shRNA scrambled (Control) or shRNA1 against ACBP (ACBP^KD^) and collected one week later for bulk mRNAseq. The graph shows normalized number of reads for ACBP in each condition (mean ± SEM, *n* = 6 samples per group, unpaired t-test ****p < 0.0001). **B** Volcano plot of RNAseq results comparing Control vs ACBP^KD^ global expression levels. **C** Gene set enrichment analysis (GSEA) of cell division-related pathways (left: Hallmark G2M Checkpoint, right: Hallmark E2F Targets) downregulated in ACBP^KD^ cells.**D** Gene Set enrichment analysis in Control vs ACBP^KD^ cells. Normalized Enrichment Score (NES) negative values indicate pathways enriched in Control cells, while positive NES values show pathways enriched in ACBP^KD^ cells. All pathways displayed have FDR q-val < 0.05.**E** tSNE plot showing primary GBM tumor cell clustering with cell type annotations [22] obtained from single-cell mRNA sequencing data available at https://gbm.cells.ucsc.edu. **F** Feature plots of ACBP and invasion-associated genes Vimentin and Ptprz1.

To have a deeper understanding on the role of ACBP in cancer cells, we interrogated the transcriptional profile of GBM cells after ACBP knockdown (Fig S1A). We performed bulk mRNA sequencing on serum-grown human GBM cell line LN229, 7 days after lentiviral delivery of scrambled control (will be further referred as Control) or ACBP shRNA1 (ACBP^KD^). First, we confirmed in our sequencing data that ACBP expression was significantly downregulated in ACBP^KD^ samples (Fig. 1A) and that Control and ACBP^KD^ groups have significant differences in their gene expression profiles (Fig. 1B) (complete list of differentially expressed genes in supplementary Table S1). In gene set enrichment analysis (GSEA [21]), we observed that G2-M checkpoint, E2F targets and other cell division-related pathways were significantly downregulated in ACBP^KD^ cells, confirming our previous observations that the lack of ACBP induces a strong decrease in human GBM cell proliferation [11] (Fig. 1C). Given that ACBP is upregulated in several cancer types (supplementary Fig S2A) (The Cancer Genome Atlas database, TCGA), we tested ACBP-sensitivity in a panel of cell lines derived from medulloblastoma, lung, colorectal and breast cancers. Notably, diverse cell lines reduced their proliferation rates upon ACBP knockdown (supplementary Fig S2B), suggesting that ACBP control over tumor growth is a common phenomenon across different cancer types. We have previously shown that ACBP-mechanism of action involves FAO [11]. Indeed, ACBP^KD^ GBM cells exhibited a higher accumulation of fluorescently labeled-palmitate, indicating a reduced utilization of fatty acids (supplementary Fig S2C). The fact that ACBP^KD^ less-responsive cell lines exhibited lower expression levels of FAO-related genes such as Acadm (Medium-chain Acyl-coenzyme A Dehydrogenase) and Cpt1-A (Carnitine palmitoyltransferase I) (supplementary Fig S2D) suggests that ACBP mechanism of action is conserved across cancer types and is likely dependent on the metabolic state of the cancer cell.

Further analysis of our GBM RNAseq data revealed a strong downregulation of genes belonging to the epithelial mesenchymal transition (EMT) pathway, cell migration, chemotaxis and cell-cell adhesion (Fig. 1D). Overall, these analyses suggested that ACBP downregulation does not only decrease proliferation but also drives GBM cells to adopt a less motile phenotype. Recent transcriptional studies at single-cell resolution allowed the classification of diverse cell types that coexist within highly heterogeneous GBM tumors [22, 23]. We explored the expression pattern of ACBP in GBM cell populations using single-cell RNAseq human GBM cell atlas [22] available at UCSC Cell Browser [24] (Fig. 1E). ACBP expression levels varied among different clusters, with the highest expression detected in neoplastic cells that exhibit molecular signatures of progenitor or immature populations and the lowest in differentiated cells (Fig. 1F). The prominent expression of ACBP both in proliferative cells (cluster 1) and in other cell populations enriched in invasion-associated genes such as Vimentin and Ptprz1 [22, 25] is in agreement with the idea of a dual role for ACBP in proliferation and invasion (Fig. 1F).

### ACBP downregulation impairs human GBM cell migration and tumor invasion

**Fig. 2:**
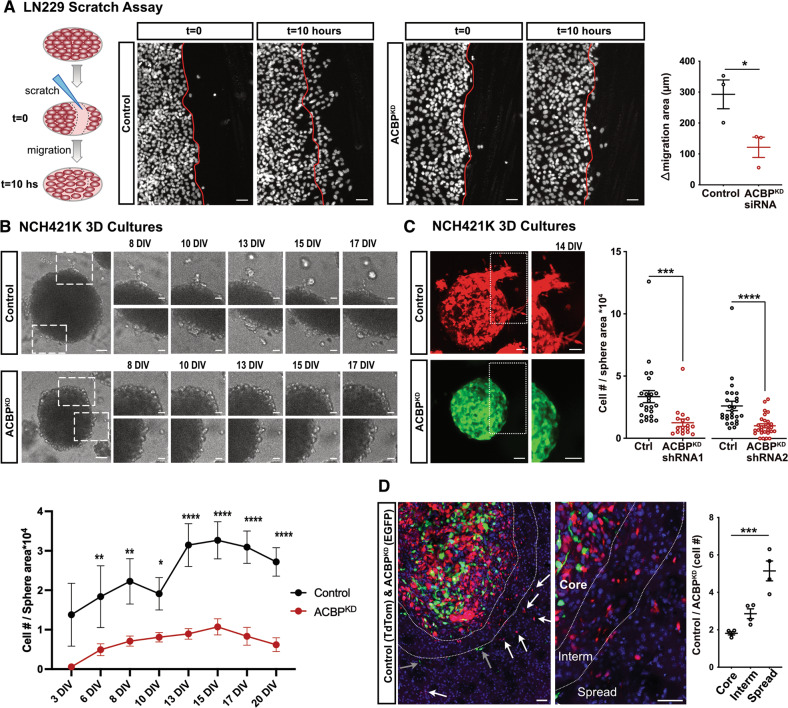
ACBP knockdown impairs GBM cell migration and invasion in vitro and in vivo. **A** Left: LN229 cells were transfected with either Control or ACBP siRNA. Upon reaching confluency, coverslips were scratched with a pipette tip to generate an empty cell area and cells were imaged every 10 min for a total of 10 h. Images show the initial and final timepoint, dashed lines indicate the border of the empty cell area at t = 0. Scale bars: 50 μm. Right: Quantification of the area covered by LN229 Control and ACBP^KD^ migrating cells after 10 h (mean ± SEM, *n* = 3 experiments per group, unpaired two-tailed t-test *p < 0.05). See also Supplementary movies 1, 2. **B** Top: NCH421k tumorspheres expressing either shRNA control or shRNA1 ACBP (ACBP^KD^) were plated in Matrigel and imaged over time at the indicated time points. Enlarged areas show examples of invasive cells exiting the spheres and invading the 3D matrix in Control conditions. Cells from ACBP^KD^ tumorspheres remained mostly stationary. Scale bars: 20 μm. Bottom: quantification of the number of invading cells, normalized by the sphere size for Control and ACBP^KD^ groups (mean ± SEM, *n* = 4–31 spheres per group, Mixed-effect analysis *p* < 0.0001, Sidak’s multiple comparisons test *p < 0.05, **p < 0.01, ****p < 0.0001). **C** Left: NCH421k tumorspheres expressing either shRNA control or shRNA1 ACBP were plated in Matrigel and imaged 14 days later. Pictures show representative examples of tumorspheres from each group. Scale bars: 50 μm. Right: Quantification of the number of cells invading the 3D matrix from Control and ACBP^KD^ tumorspheres, normalized by the sphere size for shRNA1 and shRNA2 sequences (mean ± SEM, *n* = 17–24 spheres per group, two-tailed t-test with Welch’s correction ***p < 0.001 for shRNA1, and mean ± SEM, *n* = 26–27 spheres per group, Mann-Whitney two-tailed test ****p < 0.0001 for shRNA2). **D** NSG mice were xenotransplanted with a mix of Control and ACBP^KD^ (shRNA1) NCH421K cells and sacrificed 35 days post-surgery. Left: Confocal picture of a tumor showing Control (red) and ACBP^KD^ (green) cells, overview and enlarged area. Core, intermediate and spread areas are separated with dashed lines. White and gray arrows point at Control and ACBP^KD^ invasive cells, respectively, migrating in the spread area. Scale bar: 50 µm. Right: Control/ACBP^KD^ cell ratio in core, intermediate and spread area (mean ± SEM, n = 4 tumors, one-way ANOVA with adjusted *p*-value for multiple comparisons ***p < 0.0005).

To investigate the relevance of ACBP on GBM cell migration and invasion, we began by altering the expression of ACBP in two different human GBM cell lines. First, serum-grown LN229 and second, patient-derived GBM stem-like cell line NCH421K. To evaluate the impact of ACBP downregulation on LN229 cell migration, we performed scratch assays using cells transfected with either Control or ACBP-targeting siRNA (supplementary Fig S1B). We observed a stark decrease of total area of migration in ACBP^KD^ group (Fig. 2A and supplementary movies 1 and 2). Since NCH421K are stem-like cells and are grown under serum-free conditions in suspension culture, we transduced the cells with lentiviruses encoding for either Control or ACBP shRNA1 constructs. Following lentiviral transduction and sphere formation, we embedded Control and ACBP^KD^ NCH421K spheres into Matrigel to serve as extracellular matrix (ECM) and let individual cells migrate away from the sphere surface and invade the ECM. We monitored the spheres over time and observed that while control cells were dynamic and invaded the ECM, ACBP^KD^ cells mostly remained within the spheres (Fig. 2B). A time-point quantification showed a strong decrease in the number of cells migrating away from the sphere surface in ACBP^KD^ compared to Control NCH421K cells, starting at day 6 after embedding (Fig. 2B). We confirmed the specificity of our observations by using a different ACBP shRNA sequence (shRNA2, supplementary Fig 1A). Quantifications at day 14 after embedding showed a consistent reduction in the migration capacity of patient-derived GBM 3D cultures both via shRNA1 and shRNA2 knockdown (Fig. 2C). Overall, these data demonstrate that ACBP downregulation in human GBM cells significantly impairs their ECM invasion capacity in vitro.

Next, to confirm that our in vitro findings can be translated to the in vivo setting, we transduced NCH421K cells with either Control or ACBP shRNA1 constructs expressing TdTomato or EGFP, respectively. We then mixed the two groups of cells in 1:1 ratio, orthotopically xenografted them into immunodeficient Nod scid Gamma (NSG) mice and allowed tumors to grow for 5 weeks. Histological analysis showed that in the tumor core area, Control/ACBP^KD^ cell ratio nearly doubled despite the 1:1 injection ratio, demonstrating that Control cells proliferate more and outnumber the ACBP^KD^ cells, confirming our earlier observations [11] (Fig. 1C). Importantly, the same analysis revealed that Control/ACBP^KD^ cell ratio progressively increased the further away from the tumor core (Fig. 2D). We reasoned that if Control and ACBP^KD^ cells invaded the healthy brain to similar capacities, we would obtain a similar cell number ratio in all invasion areas; however, since the ratio progressively increased, ACBP^KD^ cells likely lag behind on migration compared to Control cells. Together, our data demonstrate that ACBP downregulation impairs the migration and invasion capacity of human GBM, in vitro and in vivo.

### ACBP control on GBM migration and tumor invasion depends on fatty Acyl-CoA binding

**Fig. 3:**
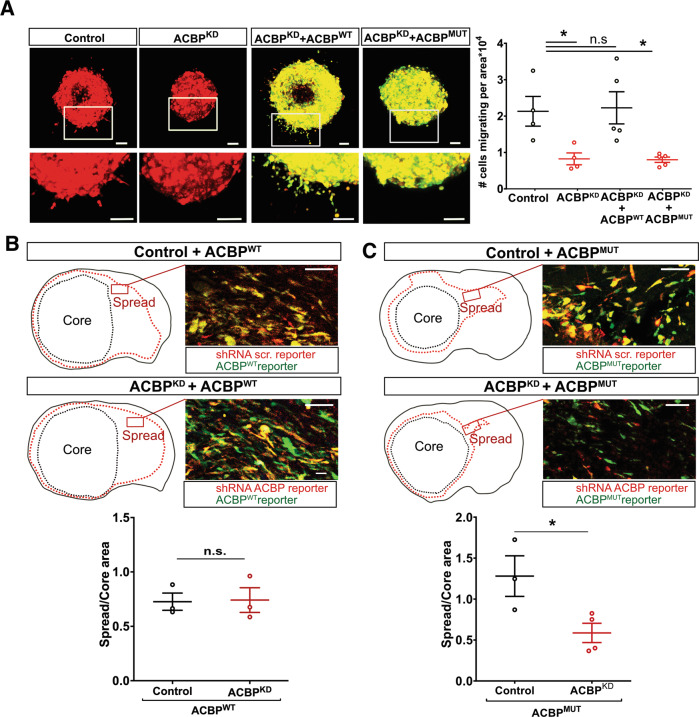
ACBP requires binding to acyl-CoA to support GBM invasion. **A** Left: Representative pictures of NCH421k tumorspheres expressing either shRNA Control (+ TdTomato), shRNA1 ACBP (ACBP^KD^ + TdTomato), ACBP^KD^ + bovine ACBP wildtype (ACBP^WT^ + EGFP) or ACBP^KD^ + bovine ACBP low binding mutant (ACBP^MUT^ + EGFP) plated in Matrigel. Boxed areas are enlarged below the respective images. Scale bars: 50 μm. Right: Quantification of the number of cells invading the extracellular matrix in each group, normalized by the sphere size (mean ± SEM, *n* = 4–5 spheres per group, one-way ANOVA with adjusted *p*-value for multiple comparisons *p < 0.05). **B** NSG mice were xenotransplanted with shRNA Control or shRNA1 ACBP^KD^ NCH421K cells co-expressing bovine ACBP^WT^. Mice were sacrificed when first moderate symptoms were observed (median survival 48 and 53 days for Control + ACBP^WT^ and ACBP^KD^ + ACBP^WT^, respectively, *n* = 4–6 mice per group, reported in [11]). Top: Outlines of representative whole brain coronal sections with core and spread tumor areas delineated with black and red dotted lines, respectively. Pictures belong to the indicated brain areas and show fluorescently-labeled tumor cells (shRNA Control and shRNA ACBP in red, ACBP^WT^ in green). Scale bars: 100 μm. Bottom: Quantification of spread/core area ratios for each experiment (mean ± SEM, *n* = 3 mice per group, unpaired two-tailed t-test *p* = 0.91). **C** NSG mice were xenotransplanted with shRNA Control or ACBP^KD^ NCH421K cells co-expressing bovine ACBP^MUT^. Mice were sacrificed when first moderate symptoms were observed (median survival 41 and 64 days for Control + ACBP^MUT^ and ACBP^KD^ + ACBP^MUT^, respectively, *n* = 4–6 mice per group, reported in [11]). Top: Outlines of representative whole brain coronal sections with core and spread tumor areas delineated with black and red dotted lines, respectively. Pictures belong to the indicated brain areas and show fluorescently-labeled tumor cells (shRNA Control and shRNA ACBP in red, ACBP^MUT^ in green). Scale bars: 100 μm. Bottom: Quantification of spread/core area ratios for each experiment (mean ± SEM, *n* = 3–4 mice per group, unpaired two-tailed t-test *p = 0.03).

ACBP binds with high affinity to medium and long chain fatty acyl-CoA (M-LCACoA) [17]. Therefore, we set out to test whether our observed migration defect after ACBP downregulation is dependent on its M-LCACoA binding function. We utilized bovine ACBP variants that are not targeted by our human ACBP shRNA sequences. A low-binding variant has a K32A mutation that does not interfere with protein folding or structure but effectively reduces (~400-fold decrease) the affinity of the protein to M-LCACoA substrates (ACBP^MUT^) [26]. In addition, it carries a M24C mutation that does not interfere with either the structure or binding dynamics of the protein. A control ACBP variant possesses only the M24C mutation (ACBP^WT^). Both variants are co-expressed with an EGFP reporter [11]. We performed rescue experiments in NCH421K spheres using either ACBP^WT^ or ACBP^MUT^ constructs to determine the contribution of ACBP binding to acyl-CoAs in GBM invasion (Fig. 3A). We observed that while control and ACBP^KD^ + ACBP^WT^ cells had comparable number of cells migrating away from sphere surface, the number of invasive cells in ACBP^KD^ + ACBP^MUT^ spheres remained low, similar to ACBP^KD^ spheres (Fig. 3A). Thus, only ACBP^WT^ and not ACBP^MUT^ rescues ACBP^KD^ phenotype. These results indicate that ACBP depends on its M-LCACoA binding function to control the ECM invasion of human GBM cells in vitro.

We next tested whether our in vitro findings would translate to the in vivo setting. We orthotopically xenografted NCH421K cells expressing either shRNA Control or ACBP^KD^ and, additionally, bovine ACBP^WT^ or ACBP^MUT^ (Fig. 3B, C). In histological analysis, we quantified the ratio of the spread area of each tumor to the area of its own core and compared it to their corresponding control groups. Our results demonstrated that while ACBP^KD^ + ACBP^WT^ tumors had comparable spread/core area ratio to Control + ACBP^WT^ group, ACBP^KD^ + ACBP^MUT^ tumors had significantly less spread/core area ratio compared to their corresponding control group, Control + ACBP^MUT^ (Fig. 3B, C). Thus, only the wild-type ACBP and not the low-binding mutant could rescue ACBP^KD^ low-spreading phenotype in vivo. Altogether, our results clearly demonstrate that both in vitro and in vivo, ACBP binding to M-LCACoA is necessary for its function in GBM tumor cell invasion.

### GBM tumor cell invasion is directly controlled by fatty acid oxidation

**Fig. 4:**
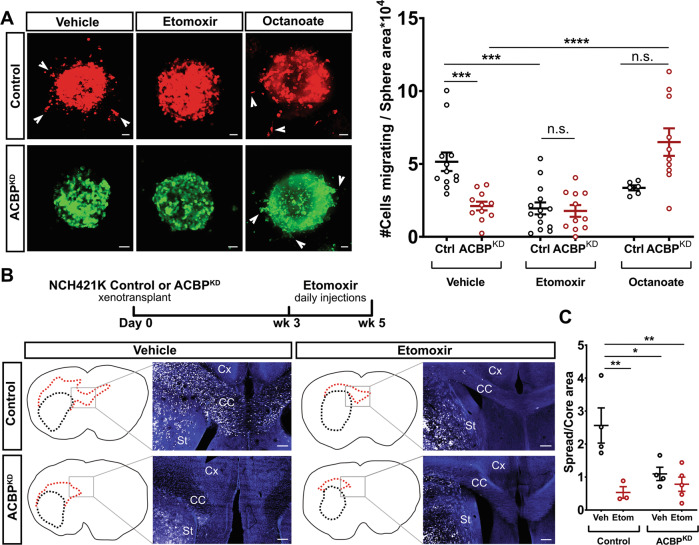
ACBP^KD^-induced invasion deficit is mimicked by inhibition of fatty acid oxidation and rescued by octanoate. **A** Left: Representative pictures of NCH421k Control (shRNA scrambled + TdTomato) and ACBP^KD^ (shRNA1 ACBP + EGFP) tumorspheres treated with vehicle, FAO-inhibitor Etomoxir (40 µM) or Octanoate (1 mM). Arrowheads point at invasive cells exiting the spheres. Scale bars: 50 μm. Right: Quantification of the number of cells invading the extracellular matrix in each group, normalized by sphere size (mean ± SEM, *n* = 6–14 spheres per group, one-way ANOVA with adjusted *p*-value for multiple comparisons ***p < 0.0005, ****p < 0.0001). **B** Top: Experimental timeline for in vivo Etomoxir injections in mice bearing either Control or ACBP^KD^ NCH421K tumors. Bottom: Outlines of representative whole brain coronal sections with core and spread tumor areas delineated with black and red dotted lines, respectively. Pictures belong to the indicated brain areas and show fluorescently-labeled tumor cells (white) and DAPI signal (blue). St: striatum, C.C.: corpus callosum, Cx: cortex. Scale bars: 200 µm. **C** Quantification of spread/core area ratios per brain in each experimental group (mean ± SEM, *n* = 3–5 mice per group, one-way ANOVA with adjusted *p*-value for multiple comparisons *p < 0.5, **p < 0.01).

Since ACBP is known to control FAO rates in GBM cells [11], and its downregulation had significantly negative impact on GBM cell migration and invasion via its binding to M-LCACoA substrates, we hypothesized that FAO might be involved in the underlying mechanism by which ACBP controls GBM invasion. To test this hypothesis, we generated Control and ACBP^KD^ NCH421K spheres, embedded them into matrigel and applied either vehicle or the pharmacological FAO-blocker Etomoxir, an inhibitor of Cpt1-A [27]. We minimized off-target effects of this drug [28, 29] by using final concentrations below 100 μM. Moreover, we complemented these experiments with a FAO-rescue approach via addition of Octanoate, a medium-chain fatty acid that can freely pass through mitochondrial membrane without the need for a transporter or fatty acid binding protein [12, 30], thereby increasing FAO rates independent of ACBP expression. We observed that Etomoxir treatment (40 μM) was detrimental for GBM cell invasion. Furthermore, Octanoate (1 mM) was able to rescue the invasion capacity of ACBP^KD^ GBM cells (Fig. 4A). Overall, these results strongly suggest that FAO is the cellular mechanism behind GBM cell invasion in vitro.

Next, we tested whether FAO controls GBM tumor invasion in vivo as well. Therefore, we orthotopically xenografted either control or ACBP^KD^ NCH421K cells to NSG mice, allowed tumors to grow for 3 weeks, treated the mice with either vehicle or Etomoxir for 2 weeks and histologically evaluated the extent of GBM invasion (Fig. 4B). We observed a significant reduction in the tumor spread/core area ratio in Control + Etomoxir group compared to Control + vehicle group (Fig. 4B, C). Importantly, Control + Etomoxir group spread/core ratio was indistinguishable from ACBP^KD^ + vehicle and ACBP^KD^ + Etomoxir groups (Fig. 4B, C). Interestingly, ACBP^KD^ tumors had a similar spread/core area ratio regardless of vehicle or Etomoxir injection, indicating that the already low rate of FAO in these tumors renders Etomoxir ineffective (Fig. 4B, C). Overall, these data suggest that ACBP controls GBM tumor invasion via FAO, both in vitro and in vivo.

### ACBP Affects GBM Cell Invasion Capacity via Itgb1 regulation

**Fig. 5:**
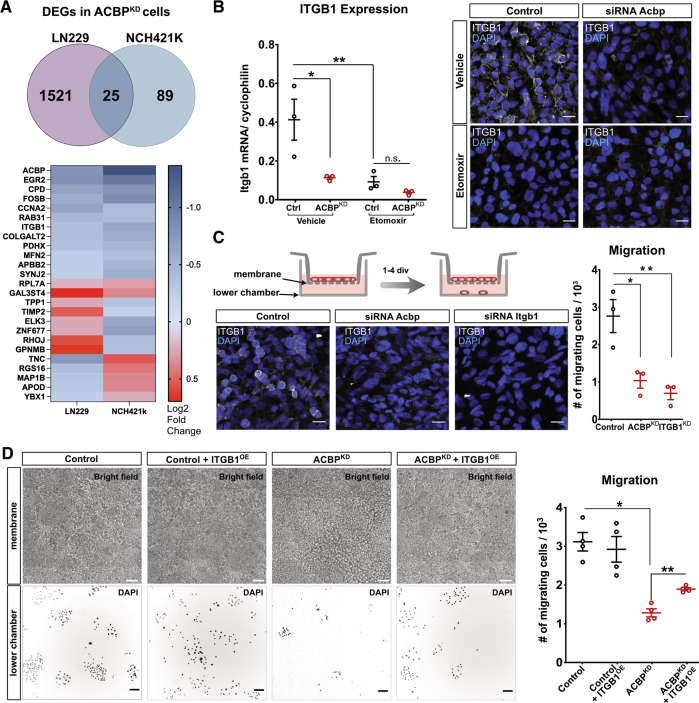
ACBP^KD^ and FAO inhibition downregulate invasion-related genes. **A** Top**:** Venn diagram of differentially expressed genes in LN229 and NCH421K ACBP^KD^ cells compared to control cells (calculated with DESeq2, *n* = 5 and 4 samples per condition for LN229 and NCH421K, respectively, adjusted *p*-value <0.01). Bottom: List of common genes differentially expressed in ACBP^KD^ cells (both cell types) with color-coded log2 Fold changes. **B** LN229 Control or ACBP^KD^ cells were treated with either vehicle or Etomoxir (40 μM) for 5 days. Left: qRT-PCR data showing Itgb1 fold expression changes (mean ± SEM, *n* = 3 samples per group, one-way ANOVA with adjusted *p*-value for multiple comparisons *p < 0.05, **p < 0.01). Right: Immunostainings showing ITGB1 protein expression in each condition (ITGB1 channel was acquired with exactly the same microscopy parameters in all cases). Scale bars: 20 μM.**C** LN229 cells were transfected with either Control, Acbp or Itgb1 siRNAs and plated at confluency for transwell assays. Pictures show representative images of ITGB1 immunostainings in each condition (ITGB1 channel was acquired with exactly the same microscopy parameters in all cases). Scale bars: 20 μM. Transwell migration assay results for Control, Acbp or Itgb1 knockdown conditions (mean ± SEM, *n* = 3 samples per group, one-way ANOVA with adjusted *p*-value for multiple comparisons). **D** LN229 cells were transfected with either Control or Acbp siRNAs and transduced with either Control or ITGB1-overexpressing lentivirus. Cells from each group were plated at confluency for transwell assays. Pictures show representative bright field images of the upper (membrane) chamber and DAPI-stained migrating cells in the lower chambers, 4 days after plating. Scale bars: 50 μM (top) and 100 μM (bottom). Transwell migration assay results for each group (mean ± SEM, *n* = 4 samples per group, one-way ANOVA with Welch’s correction, adjusted *p*-value for multiple comparisons *p < 0.05, **p < 0.01).

Next, we were interested in potential molecular factors underlying the impaired invasion capacity of ACBP^KD^ GBM tumor cells. We performed bulk mRNA sequencing of Control and ACBP^KD^ patient-derived NCH421K cells to expand our already existing gene expression dataset and find common tumor invasion targets in serum-grown and serum-free GBM cells. Differential gene expression analyses served to identify 114 and 1547 up- or down-regulated genes (adjusted *p*-value <0.01) in NCH421K ACBP^KD^ and LN229 ACBP^KD^ samples, respectively (complete list of NCH421K ACBP^KD^ differentially expressed genes in supplementary Table S2). Twenty-five of these genes were differentially expressed in both datasets (overlap *p* = 0.0001, hypergeometric test) (Fig. 5A). As expected, the top down-regulated gene from the common list was ACBP (−0.78 log2 fold change, *p* = 7.5E-42 for LN229 and −1.36 log2 fold change, *p* = 6.22E-77 for NCH421K) (Fig. 5A). As we were particularly interested in invasion-related genes, we focused our attention on Integrin beta-1 (Itgb1) [31, 32] (−0.39 log2 fold change, *p* = 5E-6 for LN229 and −0.6 log2 fold change, *p* = 2.17E-5 for NCH421K) (Fig. 5A). Since ACBP expression controls FAO in GBM cells [11] and here we show that FAO can subsequently modulate GBM tumor cell migration, we tested whether ITGB1 expression could be modulated directly by altering FAO. We administered Etomoxir or vehicle to Control and ACBP^KD^ cells for 5 days and quantified Itgb1 gene expression, both at the mRNA and protein levels. We observed a strong decrease in Itgb1 expression in Control + Etomoxir compared to Control + vehicle group, indicating that FAO modulates Itgb1 expression. However, we found no Itgb1 expression difference between ACBP^KD^ Etomoxir and ACBP^KD^ vehicle groups (Fig. 5B). We conclude that low FAO levels likely mediate the downregulation of Itgb1 in ACBP^KD^ GBM cells.

To test the relevance of ITGB1 in human GBM cell migration and compare its potential effect to that of ACBP^KD^, we downregulated ITGB1 and ACBP expression in LN229 cells using siRNA transfections and performed transwell migration assays. Our results demonstrated that both ACBP^KD^ and ITGB1^KD^ generate a substantial migration deficit in human GBM cells (Fig. 5C). To test whether ACBP and ITGB1 participate in a common pathway, we performed rescue experiments using an Itgb1-expressing lentivirus. Restoring ITGB1 expression in ACBP^KD^ cells partially rescued the migration capacity of ACBP-silenced cells in Transwell migration assays (Fig. 5D), demonstrating the involvement of this integrin in ACBP-mediated migration pathway.

## Discussion

Lipid metabolic reprogramming in GBM has received much attention in the last years, as it became evident its contribution to the pathogenesis of these aggressive tumors [8]. Our present work demonstrates that Acyl-CoA Binding Protein supports GBM invasion, in addition to proliferation [11]. Notably, both cellular effects depend on ACBP binding to acyl-CoAs, are mimicked by FAO-inhibition and can be rescued by restoring FAO levels. Thus, even though we cannot rule out that other lipid metabolism pathways might also contribute to ACBP-induced cellular effects, our data suggest that FAO is the main mediator for ACBP function in GBM. These results are in agreement with recent in silico observations indicating that highly motile tumor cells from GBM patients exhibit transcriptional profiles highly enriched in FAO-related genes [33]. Other fatty acid-binding proteins such as FABP3, FABP5, FABP6 and FABP7 have been also implicated in glioma migration or invasion [34–38]. However, these fatty acid carriers have not been related to FAO but to other lipid pathways, for example lysosomal membrane integrity in the case of FABP3 [37].

Studies in melanoma models showed that cancer cells take up adipocyte-derived exosomes that carry fatty acids and FAO enzymes. This exchange results in elevated FAO rates, which in turn induces melanoma cell migration [39]. At the cellular level, FAO-induced mitochondria activity results in the redistribution of mitochondria into invasive protrusions, a cellular rearrangement necessary to support FAO-induced cell migration [39]. Interestingly, similar mitochondrial repositioning into invadopodia structures occurs in GBM cells, resulting in increased cell invasion, possibly due to the easy accessibility of local ATP sources that fuel the energy demands of motile protrusions [40, 41]. Whether ACBP-dependent FAO levels affect mitochondria relocation also in GBM cells remains to be investigated.

We found Itgb1 among the common downregulated genes in NCH421K and LN229 ACBP^KD^ less-motile cells. Accordingly, integrin binding pathways are enriched in human GBM highly-motile cells [33]. Integrins are heterodimeric transmembrane receptors that act as adhesion molecules by binding to proteins from the extracellular matrix or neighboring cells, thereby transducing biochemical and mechanical cues to cellular responses [42]. Previous studies have related integrins to invasion and metastatic behavior in GBM and other cancer types [32, 43–45], rendering these cell-adhesive receptors as attractive targets for cancer therapy. Indeed, several integrin-inhibitors have been explored in clinical trials as anti-cancer drugs [46]. Unfortunately, the first phase 3 clinical trial using an integrin inhibitor (Cilengitide) failed to show significant benefits for GBM patients [47]. Nevertheless, the most likely explanation for the poor outcome might relate to the unfavorable pharmacokinetics of the drug and the lack of reliable biomarkers, not the target itself [48]. Our preclinical results suggest that targeting ACBP could affect ITGB1 expression levels in tumors, providing an alternative route to manage ITGB1 activity in GBM patients.

The fact that blocking FAO resulted in similar Itgb1 downregulation suggests that low Itgb1 levels observed in ACBP^KD^ cells were likely caused via reduced FAO rates instead of alternative pathways triggered in ACBP^KD^ cells. Previous studies in hepatocytes demonstrated that FAO can provide up to 90% of acetyl-carbon for histone acetylation, affecting the cell transcriptional profile by epigenetic regulation [49]. Along the same lines, generation of acetyl-CoA via FAO upregulation in breast cancer cells epigenetically regulate EMT target genes, which in turn induces a metastatic state [50]. Altogether, these results suggest that FAO contribution to cell invasion is not restricted to providing an energy source but it also involves global transcriptional changes that supports the invasive phenotype. Besides integrins, we identified other ACBP-regulated genes possibly related to cell migration such as carboxypeptidase C [51], FOSB [52] and SYNJ2 [53, 54]. The exact role of such genes in GBM invasion and whether they could provide druggable targets merits further investigation.

A classical view of GBM as mainly glycolytic tumors has led to the idea of implementing a high-fat, low-carbohydrate (ketogenic) diet as a therapeutic treatment for GBM patients [55]. However, the assumption that GBM cells rely exclusively on carbohydrates and not on fatty acids and/or ketone bodies as a source of energy has been challenged in the last years by several groups including ours [10, 11, 13, 56, 57]. Preclinical studies interrogated the efficacy of ketogenic diets for glioma patients with contrasting results [58]. A beneficial effect was observed in a few cases [59], but also ketogenic diet was shown to boost GBM growth in other reports [57]. Currently, there is not enough clinical data available to evaluate a potential therapeutic effect of this nutrition regime on GBM patients. Our study provides evidence for a pro-invasive role of the FAO pathway, supporting the view that ketogenic diet should be approached with caution in a clinical setup.

## Materials and methods

### Animals

Mice were kept in standard housing conditions in 12-hour dark/light cycles and received ad libitum food and water, following the German Animal Welfare Act regulations. All work was approved by the local animal welfare organization *Regierungspräsidium Karlsruhe*. We used the minimum number of mice required to detect statistically significant differences. Animal handling staff were certified by the Federation of European Laboratory Animal Science Associations (FELASA). NOD scid gamma mice were obtained from Jackson Laboratories and were kept in individually ventilated cages (IVCs) in a specific pathogen free (SPF) status facility.

### Cell culture, transfections, and lentiviral infections

LN229, U138, LN18, U87MG, D283Med, D341Med, Daoy, NCI-H1915, Calu-6, SW48, DLD-1, and ZR-75-1 cell lines were purchased from the American Type Culture Collection (ATCC). HCT116, T47D, and MDA-MB-231 were purchased from the Deutsche Sammlung von Mikroorganismen und Zellkulturen (DSMZ). Cells were cultured according to ATCC/DSMZ instructions. Human glioblastoma cells LN229 (recently authenticated by Multiplex human Cell line Authentication Test) were grown at 37 °C, 5% CO_2_ in DMEM containing 10% FBS. Previously described patient-derived glioblastoma stem-like cells NCH421K [60] were cultured as suspension spheroids in serum-free DMEM/Ham F-12 with 20% BIT supplement and 2mM L-Glutamax (Thermo Fischer). LN229 cells were transfected using Lipofectamine RNAiMAX according to manufacturer’s protocol (Thermo Fischer). To generate stable cell lines for Matrigel migration and xenografting experiments, dissociated NCH421K cells were transduced for 24 h with lentivirus of interest together with 8 μg/ml Polybrene (Merck) in regular culture media of respective cell line.

### Plasmid constructs, siRNAs and lentivirus production

To downregulate ACBP expression in human cells in vitro and in vivo, we used the following shRNA1 sequence: ATAGTGGCCATAGATGAACAGC and shRNA2: TTTATGTCGCCCACAGTTG. shRNA1 sequence was subcloned into both pCDH-EF1-TdTomato and pCDH-EF1-EGFP under the H1 promoter. A scrambled shRNA sequence subcloned in the same vector was used as a control for shRNA1. shRNA2 sequence was purchased as a custom-designed construct subcloned in the doxycycline-inducible pTRIPZ backbone (Horizon CAT ID: RHS4696-200704706). TRIPZ Inducible Lentiviral Negative shRNA Control (Horizon CAT ID: RHS4743) was used as a control for shRNA2. siRNA sequences for Acbp: TGCCATGAAAGCTTACAT, GCTAAAACGATTACTGAC and TAAAGAAAAAATACGGGA. siRNA sequence for Itgb1: GGATTCTCCAGAAGGTGGTTTCG. Both ACBP M24C (Wild Type) and ACBP M24C + K32A (Low-Binding) bovine ACBP variants are described in [26]. ACBP variant constructs were subcloned into lentiviral pCDH-EF1-T2A-EGFP vectors. For ITGB1 overexpression, we used EFIa-iTGB1 lentiviral vector (Addgene plasmid #115799) and pEGIP lentiviral vector (Addgene plasmid #26777). For lentivirus production, HEK293 cells were transfected with the viral backbone vector together with the VSVG and Delta-helper plasmids using a calcium phosphate mediated transfection protocol. Three days later, the viral particles were purified and concentrated by ultracentrifugation, as previously described [61].

### Bulk mRNA sequencing

RNA library preparation was performed using poly‐T beads, as described by the manufacturer (TruSeq Stranded mRNA Kit; Illumina, San Diego, CA, USA). Libraries were sequenced on a HiSeq2500 device via single‐end, 50 base‐pair reads (Illumina, HiSeq2500 HTv4, SR, dual‐indexing, 50 cycles). RNA-seq reads were aligned to hg38 using STAR aligner, expression in genes was quantified using RSEM. Differential expression analysis was done using DESeq2 [62]. Gene set enrichment analysis was performed using GSEA software [21].

### Single-cell mRNAseq data

Data was obtained from https://gbm.cells.ucsc.edu, experimental details in ref. [22].

### Proliferation assays

Various panels of cancer cells were transfected using Lipofectamine RNAiMax (Thermo Fisher) with control or ACBP-targeting siRNAs. After 3 days of transfection, cells were given either BrdU (Sigma) or EdU (Thermo Fisher) for 1 h and were fixed with 4% PFA. For staining, BrdU-employed cells were first treated with 1 M HCl for 45 min at 45 °C, washed and stained with anti-BrdU antibody. EdU-employed cells were stained directly using Invitrogen EdU Click-It kit (Thermo Fisher). ACBP^KD^ groups were also stained against ACBP and only those cells that had successfully downregulated the protein were included in the proliferation quantification.

### Scratch and transwell migration assays

For scratch assay, LN229 cells were seeded in a 6-well plate and transfected a day later using either non-targeting siRNA or a mix of three different ACBP-targeting siRNAs. 5 days post transfection, using a 200 µl pipette tip, a longitudinal scratch was made in each well and cells were labeled using Hoechst 3342 (Thermo Fischer) 2.5 mg/ml final concentration. The scratch in each well were time-lapse imaged using Zeiss Axio Observer system.

For ACBP and ITGB1 transwell migration assay, LN229 cells were seeded into a 24-well plate and the next day transfected with either control, Acbp, or Itgb1-targeting siRNAs. For rescue experiments, cells were seeded in a 12-well plate and transduced with either pEGIP (Control) or ITGB1 lentiviruses. Two days post transduction, cells were treated with 1 µg/ml puromycin (Thermo Fischer) for 2 consecutive days. One day after puromycin selection, cells were transfected with either Control or Acbp siRNAs. 4 days later, 50.000 cells were seeded into the upper chamber of a 24-well, 8 µm PET membrane transwell inserts in spheroid medium. Cells were allowed to migrate towards the lower chamber which contained the regular LN229 culture medium with 10% BSA for 1–4 days, then fixed with 4% PFA and processed for imaging and counting.

### Lipid consumption assay

LN229 cells were seeded into 24-well culture plates and next day NBD fluorophore-conjugated Palmitic acid (Avanti polar lipids) at a final concentration of 4 µM at 37 °C for 4 h was applied. Cells were then washed twice with 1xPBS and were transfected with either control or ACBP-targeting siRNAs. 3 days later, cells were fixed with 4% PFA, stained, and imaged for analysis.

### Matrigel migration assay

NCH421K cells were single cell dissociated and transduced with lentiviruses. One week post-transduction, 45 μl of media containing spheres were gently mixed with 105 μl Matrigel on ice, making a final concentration of 70% Matrigel. 30 μl drops of this mix was distributed to the chambers of glass-bottomed cell culture coverslips and incubated in 37 °C incubator for 3 min. After incubation, regular GBM stem-like cell media was applied onto each Matrigel drop. When no pharmacological assay was to be performed, regular media was replaced every 3 days until experimental endpoint. In case of drug application, the drug was refreshed together with the media every 3 days. Sphere imaging was done directly in the glass bottomed chambers using an inverted confocal microscope. For time-lapse imaging, acquisitions were taken every 2–3 days, starting from day 3 till day 20 after Matrigel embedding. Sphere imaging was done in glass bottom chambers using a spinning disk microscope.

NCH421K cells infected with lentivirus expressing doxycycline-inducible control or ACBP shRNA2 were treated with 1 µg/ml puromycin (Thermo Fischer) for 2 consecutive days. One day after puromycin selection, GBM stem-like cell media supplemented with doxycycline (10 mM final concentration, Sigma CAT# D9891) was replaced every 3 days till experimental endpoint. After one week of doxycycline treatment, NCH421K spheres were embedded in Matrigel and imaging was done 14 days later with a spinning disk microscope.

### Generation of orthotopic xenograft models

For xenotransplants, either NCH421K or LN229 cells were prepared into single cell suspension in their respective cell culture media. 8–12-week-old male NSG mice were randomized into different experimental groups, anesthetized with isofluorane inhalation and stereotaxically injected with 1 μl single cell suspension (containing 100,000 cells) on their right hemisphere using a pulled glass pipette. A small burr hole was made in the following coordinates: 1 mm anterior, 2 mm lateral to bregma, and through the burr hole the glass pipette was inserted 3 mm deep for injection. Mice were killed either at specific time-points before the appearance of symptoms (Figs. 2 and 4) or at ethical endpoints defined by the appearance of symptoms of moderate severity, as stated by the local animal welfare authorities (Fig. 3). The investigators were not blinded to the group allocation.

### Etomoxir injections

(R)-( + )-Etomoxir sodium salt (Tocris Biosciences) was dissolved in water and injected 15 mg/kg i.p. daily for 2 weeks along with water injection controls.

### Immunostainings and antibodies

Immunofluorescence stainings of 4% paraformaldehyde (PFA)-fixed cultured cells were performed in 24 well plates. Immunohistochemical stainings of brain tissues were performed using free-floating sections. Briefly, anesthetized mice were intracardially perfused with 4% PFA in PBS. The brains were removed, fixed overnight in 4% PFA and cut in a Leica VT 1000 S vibratome in 50 μm slices. The slices were permeabilized and blocked using 5% BSA, 0.1% Triton PBS solution for 2 h and were next incubated with the primary antibody using 3% BSA, 0.1% Triton solution overnight. The next day the sections were washed with PBS 3 times and were incubated with the secondary antibody for 4 h in 3% BSA, 0.1% Triton solution. Sections were next washed 3 times with PBS and incubated with DAPI solution for 10 min, washed once again and mounted. Antibodies used were: mouse anti-BrdU (BD Biosciences, cat# 347580), rabbit anti-ACBP (Santa Cruz cat# sc30190), chicken anti-EGFP (Abcam, cat# ab13970), rabbit anti-DsRed (Clontech Living Colors, cat# 632496), mouse anti-ITGB1 (Abcam, cat# ab24693), Alexa 647 conjugated donkey anti-mouse and anti-rabbit (Thermo Fisher, cat# A21447, cat# A31573), Alexa 488 conjugated donkey anti-chicken (Jackson ImmunoResearch Laboratories cat# 703-545-155) and Cy3 conjugated donkey anti-rabbit (Jackson ImmunoResearch Laboratories cat# 711-165-152).

### Microscopy

Live cell imaging, Matrigel migration assays, and tissue sections were imaged in Zeiss Axio Observer Widefield, Zeiss LSM 780 Spinning Disk, Zeiss LSM 710 ConfoCor 3 or Zeiss LSM 700 systems.

### Quantification and statistical analysis

For all tumor section quantifications at least 3 sections per mouse were used. For all in vitro experiments at least 3 independent viral transductions or plasmid transfections were performed and analyzed. Sample size and exact statistical test employed for each experiment are noted in Figure Legends. All statistical analyses were carried out using GraphPad Prism software. Each dataset was first tested to evaluate if the values were normally distributed (Shapiro-Wilk normality test) and whether variances between groups were comparable (F test to compare variances). Datasets were subsequently analyzed with either parametric or non-parametric tests, with or without Welch’s correction.

## Supplementary information

Supplementary Figure 1

Supplementary Figure 2

Supplementary movie Legends

Movie 1

Movie 2

Supplementary Table 1

Supplementary Table 2

Checklist

## Data Availability

All data generated for this study are available from the corresponding author (JA) upon reasonable request.
