# Supplementary material for: Targeting fatty acid oxidation via Acyl-CoA binding protein hinders glioblastoma invasion
Source: Cell Death Dis. 2023 Apr 29;14(4):296. doi: 10.1038/s41419-023-05813-0 (PMC10148872; doi:10.1038/s41419-023-05813-0)
Supplement: Supplementary file 1 — Supplementary Figure 1 [file 41419_2023_5813_MOESM1_ESM.pdf]

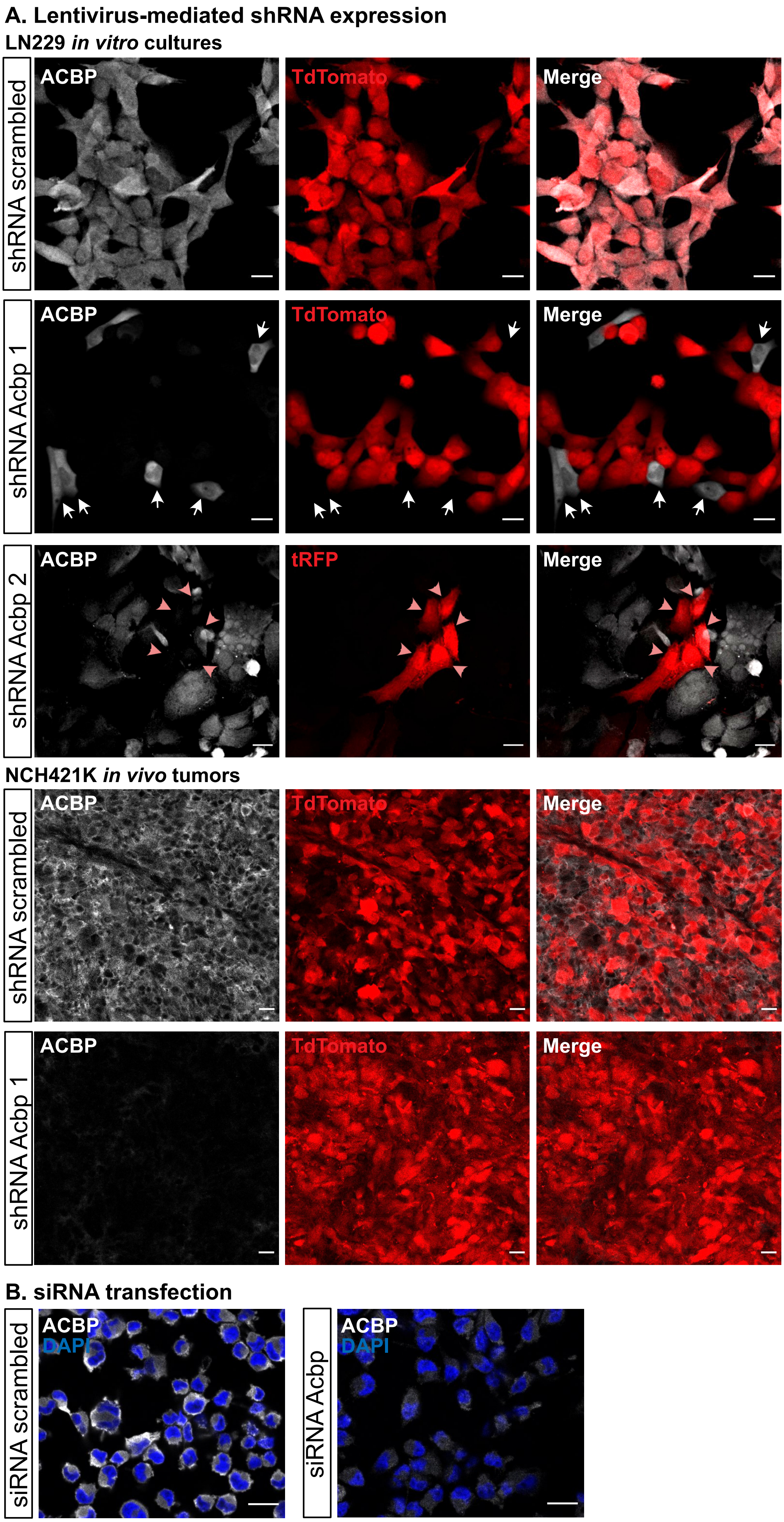
A. Lentivirus-mediated shRNA expression
LN229 in vitro cultures
shRNA scrambled
ACBP
TdTomato
Merge
shRNA Acbp 1
ACBP
TdTomato
Merge
shRNA Acbp 2
ACBP
tRFP
Merge
NCH421K in vivo tumors
shRNA scrambled
ACBP
TdTomato
Merge
shRNA Acbp 1
ACBP
TdTomato
Merge
B. siRNA transfection
siRNA scrambled
ACBP
DAPI
siRNA Acbp
ACBP
DAPI

Supplementary Figure S1. ACBP protein expression after knockdown.

A Top: LN229 cells were transduced with lentiviruses expressing either scrambled shRNA, Acbp shRNA1 or Acbp shRNA2. All lentiviruses express in addition TdTomato or tRFP. Acbp shRNA2 expression is inducible upon doxycycline administration (pTRIPZ). Pictures show representative stainings for ACBP and the fluorescent marker. White arrows in Acbp shRNA1 row point at non-infected ACBP-positive cells (lentivirus-infected red cells do not show ACBP signal). Pink arrows in Acbp shRNA2 row point at lentivirus-infected cells after doxycycline administration (red), negative for ACBP. Scale bars: 20 μM. **Bottom:** Patient-derived NCH421K cells were transduced with lentiviruses expressing either scrambled shRNA or Acbp shRNA1 and xenotransplanted in mice brains. Panels show representative pictures of tumors from each group stained for ACBP and the fluorescent marker TdTomato (both channels were acquired with exactly the same microscopy parameters in all cases). Scale bars: 20 μM.

B LN229 cells were transfected with either siRNA scrambled or siRNA Acbp and stained for ACBP plus DAPI 3 days after transfection (ACBP channel was acquired with exactly the same microscopy parameters in both cases). Scale bars: 20 μM.
