# Supplementary material for: Targeting fatty acid oxidation via Acyl-CoA binding protein hinders glioblastoma invasion
Source: Cell Death Dis. 2023 Apr 29;14(4):296. doi: 10.1038/s41419-023-05813-0 (PMC10148872; doi:10.1038/s41419-023-05813-0)
Supplement: Supplementary file 2 — Supplementary Figure 2 [file 41419_2023_5813_MOESM2_ESM.pdf]

Figure S2

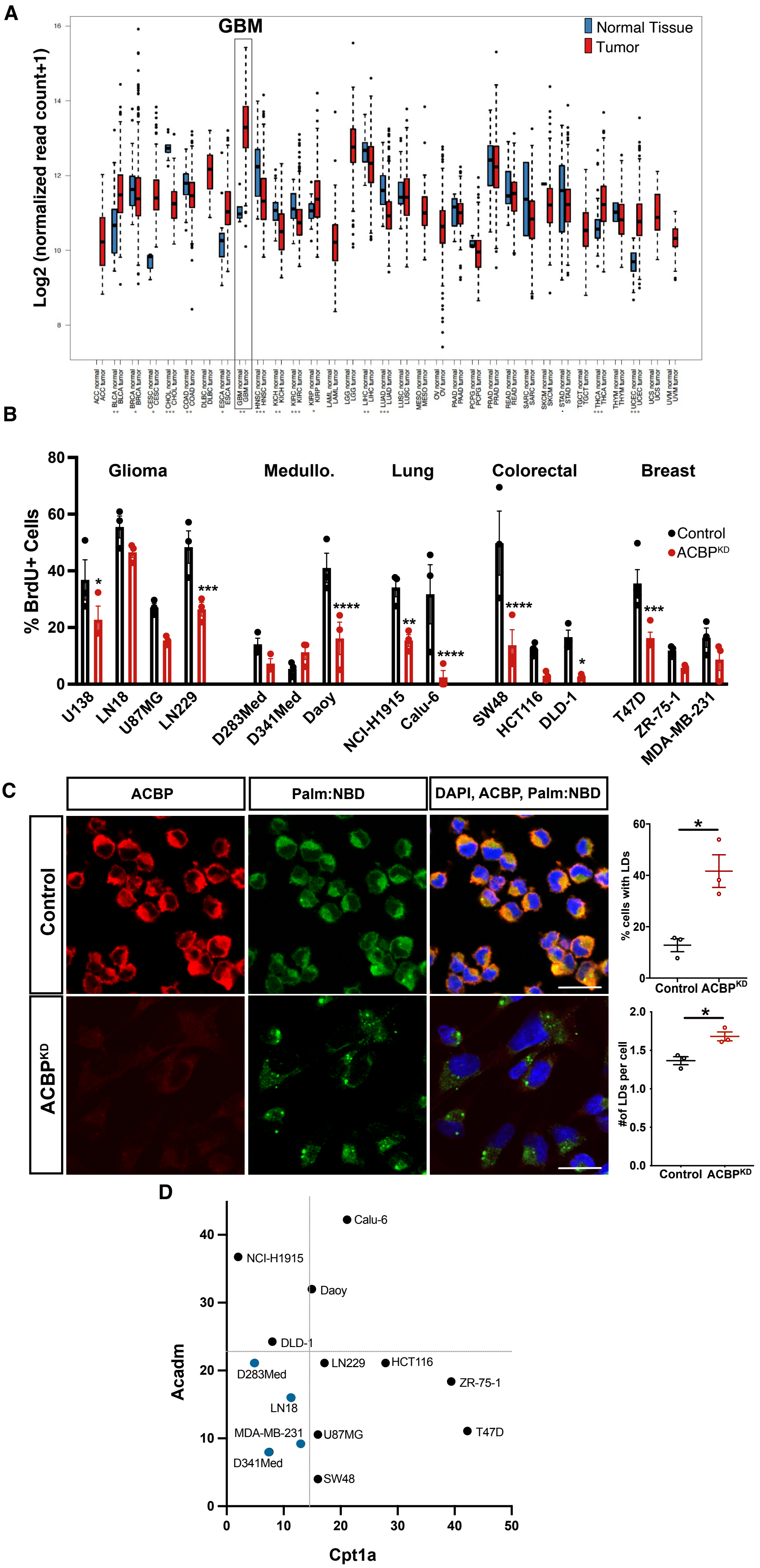
A
GBM
Normal Tissue
Tumor
Log2 (normalized read count+1)
B
% BrdU+ Cells
Glioma
Medullo.
Lung
Colorectal
Breast
Control
ACBP^KD
U138
LN18
U87MG
LN229
D283Med
D341Med
Daoy
NCI-H1915
Calu-6
SW48
HCT116
DLD-1
T47D
ZR-75-1
MDA-MB-231
C
ACBP
Palm:NBD
DAPI, ACBP, Palm:NBD
Control
ACBP^KD
% cells with LDs
#of LDs per cell
Control ACBP^KD
D
Acadm
Cpt1a
Calu-6
NCI-H1915
Daoy
DLD-1
D283Med
LN229
HCT116
ZR-75-1
LN18
MDA-MB-231
U87MG
T47D
D341Med
SW48

Supplementary Figure S2. ACBP knockdown affects lipid distribution and reduces proliferation in cell lines from different tumor types.

A ACBP/DBI expression in tumors and normal tissue from TCGA studies (Wilcoxon test with adjusted p-values: ·$p<0.1$, *$p<0.01$, **$p<0.001$, ***$p<0.0001$).

B Quantification of the percentage of BrdU+ cells in the indicated cell lines transfected with either shRNA scrambled (Control) or shRNA against ACBP ($ACBP^{KD}$) (mean ± SEM, n= 3 samples per group, two-way ANOVA *$p<0.05$, **$p<0.005$, ***$p<0.001$, ****$p<0.0001$).

C Control or $ACBP^{KD}$ LN229 cells were incubated with fluorescently labeled (NBD) palmitic acid (4μM) and fixed four days later. **Left:** Representative pictures of Control or $ACBP^{KD}$ LN229 stained for ACBP (red), NBD-palmitic acid (green) and DAPI (blue). **Right:** Quantification of percentage of cells with lipid droplets (LD) and number of LDs per cell in Control and $ACBP^{KD}$ LN229 cells (mean ± SEM, n= 3 samples per group, unpaired two-tailed t-test *$p<0.05$). Scale bars: 20 μM.

D The plot shows mRNA expression data for Acadm and Cpt1a genes from different cell lines assayed in (A). Data obtained from Cancer Cell Line Encyclopedia. ACBP low-responsive cell lines are labeled in blue.
