# Supplementary material for: Targeting fatty acid oxidation via Acyl-CoA binding protein hinders glioblastoma invasion
Source: Cell Death Dis. 2023 Apr 29;14(4):296. doi: 10.1038/s41419-023-05813-0 (PMC10148872; doi:10.1038/s41419-023-05813-0)
Supplement: Supplementary file 3 — Supplementary movie Legends [file 41419_2023_5813_MOESM3_ESM.docx]

**Supplementary movie 1. Control LN229 GBM cells migrating.**

LN229 cells were transfected with Control siRNA. Upon reaching confluency (day 6 post-siRNA transfection), coverslips were scratched with a 200µl pipette tip to generate an empty area. Nuclei were stained using HOECHST 33342, and were imaged every 10 minutes for a total of 10 hours.

**Supplementary movie 2. ACBP^KD^ LN229 GBM cells migrating.**

LN229 cells were transfected with ACBP-targeting siRNA. Upon reaching confluency (day 6 post-siRNA transfection), coverslips were scratched with a 200µl pipette tip to generate an empty area.  Nuclei were stained using HOECHST 33342, and were imaged every 10 minutes for a total of 10 hours
